# Diagnostic and Prognostic Implications of FGFR3^high^/Ki67^high^ Papillary Bladder Cancers

**DOI:** 10.3390/ijms19092548

**Published:** 2018-08-28

**Authors:** Mirja Geelvink, Armin Babmorad, Angela Maurer, Robert Stöhr, Tobias Grimm, Christian Bach, Ruth Knuechel, Michael Rose, Nadine T. Gaisa

**Affiliations:** 1 Institute of Pathology, RWTH Aachen University, Pauwelsstrasse 30, 52074 Aachen, Germany; mirja.geelvink@rwth-aachen.de (M.G.); armin.babmorad@rwth-aachen.de (A.B.); amaurer@ukaachen.de (A.M.); rknuechel-clarke@ukaachen.de (R.K.); mrose@ukaachen.de (M.R.); 2 Institute of Pathology, University Hospital Erlangen, Friedrich-Alexander University Erlangen-Nürnberg (FAU), 91054 Erlangen, Germany; Robert.Stoehr@uk-erlangen.de; 3 Department of Urology, Ludwig Maximilian University Munich, 81377 Munich, Germany; Tobias_Grimm@med.uni-muenchen.de; 4 Department of Urology, RWTH Aachen University, 52074 Aachen, Germany; chbach@ukaachen.de

**Keywords:** FGFR3, Ki67, TP53, bladder cancer, prognosis

## Abstract

Prognostic/therapeutic stratification of papillary urothelial cancers is solely based upon histology, despite activated FGFR3-signaling was found to be associated with low grade tumors and favorable outcome. However, there are FGFR3-overexpressing tumors showing high proliferation—a paradox of coexisting favorable and adverse features. Therefore, our study aimed to decipher the relevance of FGFR3-overexpression/proliferation for histopathological grading and risk stratification. *N* = 142 (*n* = 82 pTa, *n* = 42 pT1, *n* = 18 pT2-4) morphologically G1–G3 tumors were analyzed for immunohistochemical expression of FGFR3 and Ki67. Mutation analysis of *FGFR3* and *TP53* and FISH for *FGFR3* amplification and rearrangement was performed. SPSS 23.0 was used for statistical analysis. Overall FGFR3^high^/Ki67^high^ status (*n* = 58) resulted in a reduced ∆mean progression-free survival (PFS) (*p* < 0.01) of 63.92 months, and shorter progression-free survival (*p* < 0.01; mean PFS: 55.89 months) in pTa tumors (*n* = 50). *FGFR3*^mut^/*TP53*^mut^ double mutations led to a reduced ∆mean PFS (*p* < 0.01) of 80.30 months in all tumors, and *FGFR3*^mut^/*TP53*^mut^ pTa tumors presented a dramatically reduced PFS (*p* < 0.001; mean PFS: 5.00 months). Our results identified FGFR3^high^/Ki67^high^ papillary pTa tumors as a subgroup with poor prognosis and encourage histological grading as high grade tumors. Tumor grading should possibly be augmented by immunohistochemical stainings and suitable clinical surveillance by endoscopy should be performed.

## 1. Introduction

Bladder cancer is the second most common genitourinary malignancy [1]. At primary diagnosis, most of the tumors are papillary non-invasive cancers (pTa) which are mostly well differentiated but show a high rate of recurrence. Those tumors are characterized by certain molecular alterations as for example FGFR3 activation [2,3,4,5]. Up to 30% of all patients have invasive disease at diagnosis. These tumors frequently derive from flat carcinoma in situ (CIS) of the urothelium (a high grade lesion, often *TP53*-mutated) and quickly develop muscle-invasion and metastasis [6,7]. Current prognostic and therapeutic stratification in urothelial cancers is therefore based on tumor staging and grading at histological examination. Staging criteria is the depth of invasion defined by the tumor node metastasis (TNM)-classification of the Union Internationale Contre le Cancer (UICC) [8]. The tumor grading is based upon architectural order and nuclear shape features, which have been thoroughly defined as diagnostic criteria in the 2004 WHO classification of bladder cancer in order to achieve reproducible and comparable diagnoses worldwide. Low grade (LG) tumors show uniform, slightly enlarged nuclei in an orderly, polarized architecture, sometimes with a prominent palisading of the basal layer. Mitotic figures are infrequent [9,10]. High grade (HG) tumors show more pleomorphic nuclei with multiple mitotic features and various extent of architectural disarray [10]. Based on previous genetic analyses and clinical observations, it has been proposed that the histological appearance (grading) of tumors correlates with the underlying genetic alterations, and low grade tumors were regarded genetically stable, whereas high grade tumors, harboring a high number of genetic alterations, were considered genetically “unstable” [7]. Proposed prognostic markers in papillary non-invasive tumors have been the Ki67 labeling index (marker for cell proliferation) and keratin 20 expression (marker for cell differentiation). Tumors with Ki67 ≥ 15% were regarded as highly proliferative [11,12,13] and aberrant expression of keratin 20 was linked to disease recurrence in pTa tumors [14]. Lately, Hurst et al. conducted a comprehensive molecular study on *n* = 141 papillary non-invasive bladder cancers (low grade, G1 and G2 according to WHO 1973) and found lower overall mutation rates, but more mutations in chromatin modifying genes than in muscle-invasive bladder cancer, and two distinct genomic subgroups of tumors (genomic subtype 1 and 2). The majority of tumors with genomic subtype 1 showed no or only few copy-number alterations. Genomic subtype 2 was characterized by loss of 9q (including the mTORC1 regulator TSC1), increased Ki67 labeling index, upregulated mTORC1 signaling (comprising the overrepresentation of genes in processes that are involved in the unfolded protein response, glycolysis, and cholesterol homeostasis) as well as enrichment for DNA repair and cell-cycle genes [15]. *FGFR3* mutations were not found to be significantly different in both subgroups (72% vs. 89%) and *TP53* mutations were absent [15]. The authors did not show a correlation of molecular profiles with specific histological features.

However, in routine histological diagnostics, pathologists often see papillary non-invasive tumors with quite uniform, relatively small nuclei, which give a “crowded” impression, but seem to be of “low nuclear grade”. Interestingly, Ki67 labeling in these tumors is often enhanced and from this point of view a reconsideration of a possible “high grade”-biology is implicated. Opposite to the negative predictive impact of a high Ki67 index, these tumors often show a strong expression of FGFR3, which indicates an activation of the signaling pathway resulting in cellular proliferation, but is generally associated with a benign course of disease with higher recurrence rates but less progression [7]. Being aware of this diagnostic-biological “dilemma”, we delineated in this study the immunohistochemical and genetic basis of such FGFR3^high^/Ki67^high^ papillary bladder cancers in order to reveal their prognostic impact.

## 2. Results

### 2.1. Immunohistochemical Combination of Ki67-Index and FGFR3 Levels Defines Worse Patients’ Outcome

**Figure 1:**
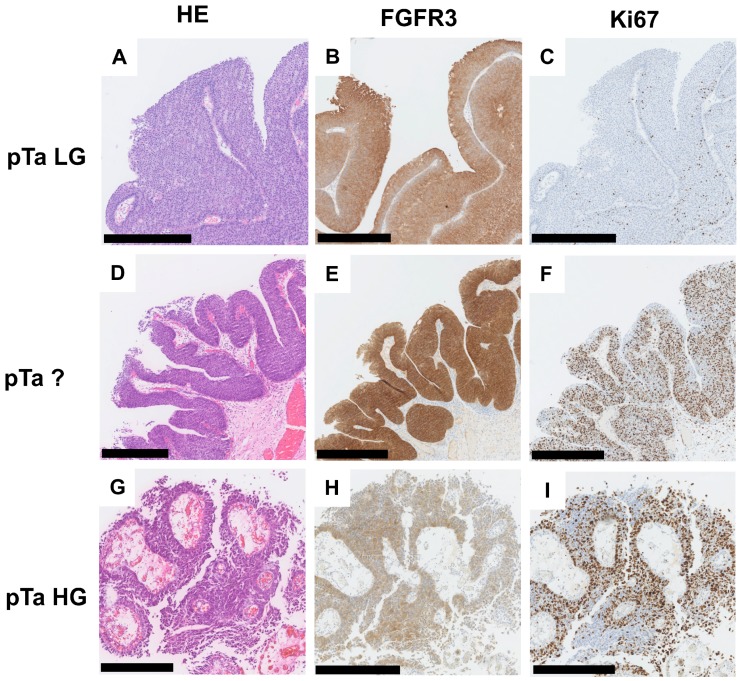
FGFR3 and Ki67 protein expression in papillary non-invasive (pTa) bladder tumors. Immunohistochemical staining for FGFR3 and Ki67 protein of representative tumors are shown. (**A**–**C**) pTa low grade (LG) tumor: (**A**) Hematoxylin and Eosin (HE) staining; (**B**) strong FGFR3 immunoreactivity; and (**C**) only a few cell nuclei are positive for Ki67 expression. (**D**–**F**) pTa tumor with “crowded low nuclear grade” (pTa?) morphology: (**D**) HE staining; (**E**) strong FGFR3 immunoreactivity; and (**F**) high nuclear Ki67 protein staining. (**G**–**I**) pTa high grade (HG) tumor: (**G**) HE staining; (**H**) moderate FGFR3 protein expression; and (**I**) high nuclear Ki67 staining. Scale bar: 500 µm; original digital magnifications vary from 5× to 7×.

Overall, FGFR3 and Ki67 protein expression was analyzed by immunohistochemistry (Figure 1A–I) in *n* = 142 primary bladder tumors comprising *n* = 82 papillary non-invasive tumors (for cohort characteristics, see Table S1). In this cohort, 87/142 patients (61.3%) showed a high Ki67-index (≥15% positivity) and 100/142 bladder cancer patients (70.4%) were characterized by strong FGFR3 expression (Tomlinson Score 3) (Figure S1A,C). In papillary non-invasive pTa tumors, 82.9% showed strong FGFR3 and 54.9% increased Ki67 expression (Figure S1B,D).

Next, associations between clinico-pathological characteristics and both FGFR3 and Ki67 protein expression were tested. FGFR3 expression and Ki67 index correlated with tumor grading (FGFR3: *p* < 0.001, Ki67: *p* < 0.001), but only FGFR3 expression was significantly associated with tumor stage (FGFR3: *p* < 0.001) (Table 1 and Table 2). No association was found between FGFR3/Ki67 and age at diagnosis or gender.

**Figure 2:**
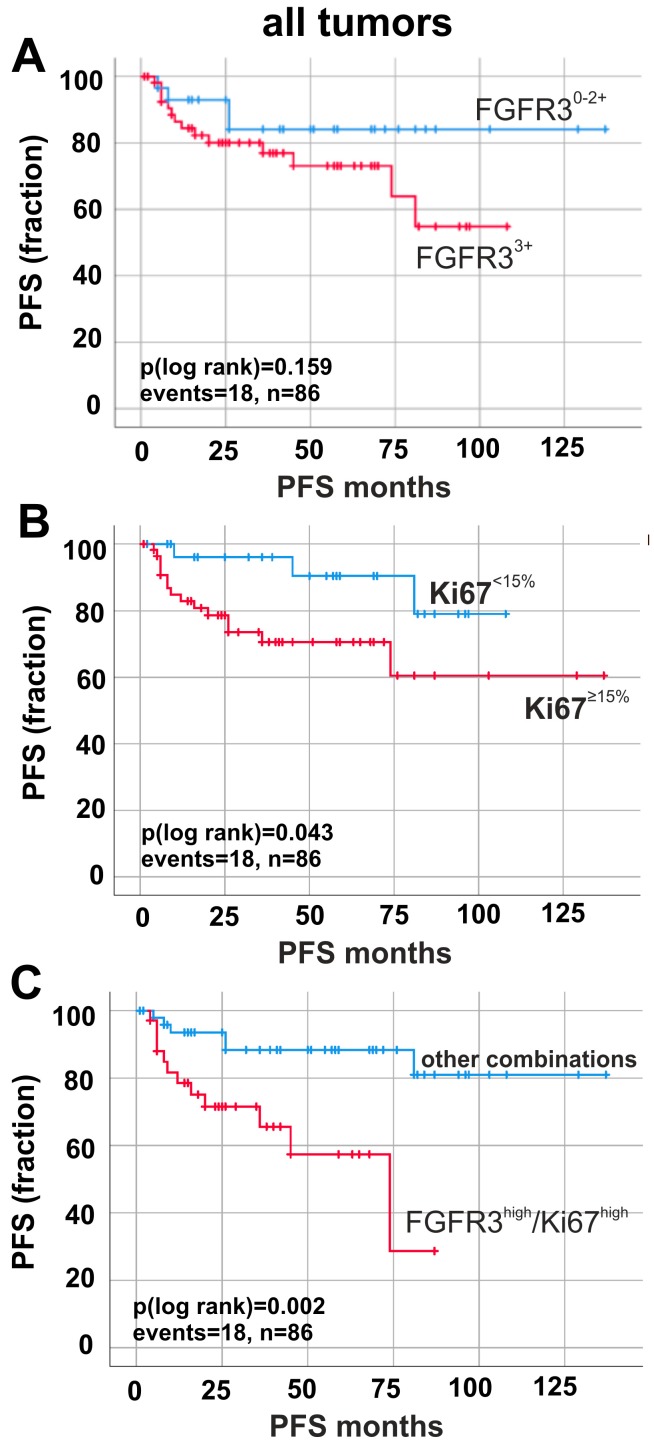
Prognostic impact of FGFR3 and Ki67 protein expression in all tumors (pTa, pT1 and pT2–4). Kaplan–Meier survival curves display progression-free survival (PFS). (**A**) Survival curves of patients with high FGFR3 expression (red curve, *n* = 56) compared to low FGFR3 expression (blue curve, *n* = 30). (**B**) Kaplan–Meier analysis of patients with high Ki67 expression (red curve, *n* = 56) compared to low Ki67 expression (blue curve, *n* = 30). (**C**) Survival curve analysis of FGFR3^high^/Ki67^high^ expression (red curve, *n* = 34) compared to all other combinations of FGFR3 and Ki67 expression (blue curve, *n* = 32). *n*: overall number of cases; events: overall events of tumor progression.

To assess the clinical impact, Kaplan–Meier analyses were performed. FGFR3 expression had no significant impact on progression-free survival (PFS) (Figure 2A). In contrast, enhanced Ki67 expression (≥15%) significantly predicted shorter progression-free survival (∆mean PFS: 2.71 months, *p* = 0.043). Finally, we aimed to decipher the potential prognostic impact of combined FGFR3 expression and Ki67 index: FGFR3^high^/Ki67^high^ status was found in *n* = 58 cases. A combined analysis of FGFR3/Ki67 positivity (Figure 2C and Figure S2A) resulted in a reduced ∆mean PFS (*p* < 0.01) of 63.92 months when comparing FGFR3^high^/Ki67^high^ tumors (mean PFS: 54.87 months ± 6.73; 95% CI: 41.78 to 68.05) with all other combinations (mean PFS: 118.78 months ± 6.95; 95% CI: 105.17 to 132.40). If, for example, both markers were expressed at low levels, bladder cancer patients showed no progressive disease at all (Figure S2A). Therefore, our results identify FGFR3^high^/Ki67^high^ tumors as an aggressive subgroup.

The calculated Cox regression model (including the potentially prognostic parameters stage, grade, age, keratin 20 and keratin 5/6) confirmed independency of the clinical impact of a FGFR3^high^/Ki67^high^ status on progression-free survival. Patients displaying a combined overexpression of FGFR3 and Ki67 showed an approximately four-fold higher risk for tumor progression (multivariate hazard ratio (HR): 3.943, 95% CI: 1.247 to 12.466, *p* = 0.019) (Table 3).

### 2.2. Prognostic Impact of Ki67-Index and FGFR3 Overexpression in Papillary Non-Invasive (pTa) Tumors

**Figure 3:**
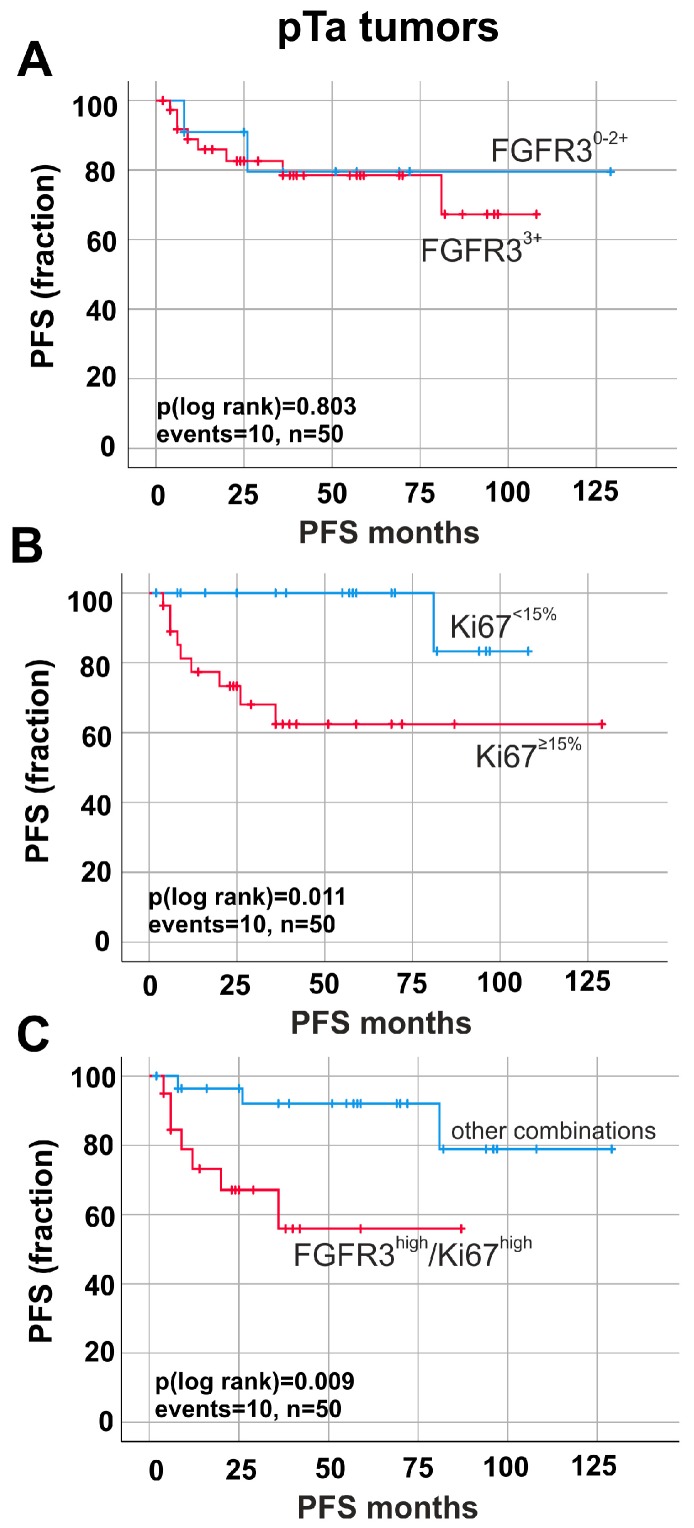
Prognostic impact of FGFR3 and Ki67 protein expression in papillary non-invasive (pTa) tumors. Kaplan–Meier survival curves demonstrate progression-free survival (PFS). (**A**) Survival curves of patients with high FGFR3 expression (red curve, *n* = 39) compared to low FGFR3 expression (blue curve, *n* = 11). (**B**) Kaplan–Meier analysis of patients with high Ki67 expression (red curve, *n* = 28) compared to low Ki67 expression (blue curve, *n* = 22). (**C**) Impact of combined markers on risk stratification of tumor progression is shown. Survival curve analysis of FGFR3^high^/Ki67^high^ expression (red curve, *n* = 20) compared to all other combinations of FGFR3 and Ki67 expression (blue curve, *n* = 30) in pTa tumors. n, overall number of cases; events, overall events of tumor progression.

Stratifying our cohort by invasiveness, i.e., into papillary non-invasive (pTa) and invasive tumors (pT1–pT4), FGFR3 overexpression (Tomlinson Score 3) was not associated with tumor progression in pTa bladder cancer (*p* > 0.05 for PFS) (Figure 3A).

Single marker analysis of high Ki67-index correlated with progression-free survival (∆mean PFS: 17.06 months, *p* = 0.011) (Figure 3B). Now, combining the two immunohistochemical markers, univariate Kaplan–Meier curve revealed a significant impact of FGFR3^high^/Ki67^high^ expression on patients’ outcome only in pTa tumors. In fact, patients with high FGFR3^high^/Ki67^high^ showed a significantly (*p* < 0.01) shorter progression-free survival (mean PFS: 55.89 months ± 9.23; 95% CI: 37.82 to 73.98) compared to those patients with all other combinations of FGFR3/Ki67 expression (mean PFS: 113.85 months ± 8.12; 95% CI: 97.94 to 129.77, *p* = 0.009) (Figure 3C).

### 2.3. Altered Molecular FGFR3/TP53 Status Predicts Worse Patients’ Survival

**Figure 4:**
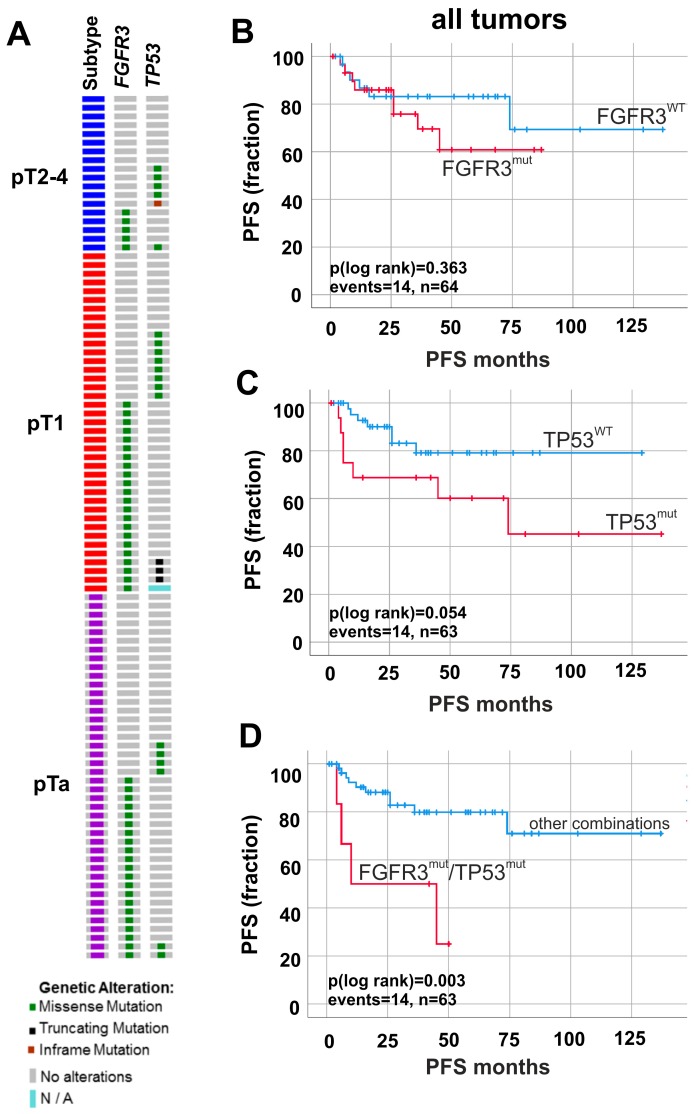
*FGFR3* and *TP53* mutation frequency and prognostic impact on tumor progression. (**A**) Oncoprint graph for *FGFR3* and *TP53* mutation analysis. (**B**–**D**) Kaplan–Meier survival curves display progression-free survival (PFS). (**B**) Survival curves of tumors with detected *FGFR3* mutations (red curve, *n* = 30) compared to non-mutated *FGFR3* gene status (blue curve, *n* = 34). (**C**) Kaplan–Meier analysis of tumors with mutated *TP53* (red curve, *n* = 17) compared to wildtype *TP53* (blue curve, *n* = 46). (**D**) Impact of double mutations on risk stratification of tumor progression is demonstrated. Univariate analysis of double mutations (red curve, *n* = 6) compared to all other combinations of mutated and non-mutated *FGFR3* and *TP53* genes (blue curve, *n* = 57). *n*, overall number of cases; events, overall events of tumor progression.

Since we hypothesized that FGFR3-overexpression and high cell proliferation might indicate a higher risk for progression in papillary non-invasive tumors, we further investigated the molecular status of our cohort by studying both mutations for *FGFR3* as papillary and *TP53* as invasive markers (for detailed mutation data, see Table S2). In total, 48 out of 99 (48.5%) analyzed patients harbored mutations within the *FGFR3* gene (Figure 4A).

The most frequent mutation was p.S249C (pTa: 13/21, pT1: 10/22, pT2–4: 2/5). *FGFR3* mutations showed no significant association with clinico-pathological parameters like tumor stage or grade (Table S3). *TP53* mutations were present in *n* = 23/98 (23.5%) patients (Figure 4A). There were *n* = 18/23 (78.3%) tumors which solely showed missense mutations (pTa: 6/6, pT1: 7/11, pT2–4: 5/6) and *n* = 5/23 (21.7%) tumors with mutations leading to a premature transcription stop either due to the appearance of a stop codon or a frameshift (pTa: 0/6, pT1: 4/11, pT2–4: 1/6). *TP53* mutations correlated with tumor grade (*p* < 0.05) but not with stage (Table S4). Mutations in both genes (referred to as double mutations) were found in *n* = 6/99 (6.1%) patients.

Survival analysis revealed no significant association between single mutations, i.e., *FGFR3* or *TP53*, with patient’s outcome for PFS (Figure 4B,C). However, mutations in both genes (*FGFR3*^mut^/*TP53*^mut^) predicted unfavorable prognosis for PFS. Double mutations led to a reduced ∆mean PFS (*p* < 0.01) of 80.30 months: *FGFR3*^mut^/*TP53*^mut^ tumors (mean PFS: 27.08 months ± 8.41; 95% CI: 10.59 to 43.57) showed shorter PFS in contrast with all other combinations (mean PFS: 107.83 months ± 8.62; 95% CI: 90.49.17 to 124.28) (Figure 4D and Figure S2B).

Multivariate analysis confirmed the prognostic impact of *FGFR3*^mut^/*TP53*^mut^ tumors. Double mutated tumors exhibited a 6.6 times higher risk for tumor progression (multivariate hazard ratio (HR): 6.563, 95% CI: 1.694 to 25.425, *p* = 0.006) (Table 4).

### 2.4. Prognostic Impact of FGFR3 and TP53 Mutations in Papillary Non-Invasive (pTa) Tumors

Next, we focused on pTa tumors, in particular those with FGFR3-overexpression and high cell proliferation. In pTa tumors, the following distribution was found: *n* = 17/42 (40.5%) *FGFR3*^wt^/*TP53*^wt^, *n* = 19/42 (45.2%) *FGFR3*^mut^/*TP53*^wt^, *n* = 4/42 (9.5%) *FGFR3*^wt^/*TP53*^mut^ and *n* = 2/42 (4.8%) *FGFR3*^mut^/*TP53*^mut^. On the contrary, pT1 tumors showed *n* = 9/39 (23.1%) *FGFR3*^wt^/*TP53*^wt^, *n* = 19/39 (48.7%) *FGFR3*^mut^/*TP53*^wt^, *n* = 8/39 (20.5%) *FGFR3*^wt^/*TP53*^mut^ and *n* = 3/39 (7.7%) *FGFR3*^mut^/*TP53*^mut^. pT2–4 tumors represented with the following mutational pattern: *n* = 8/18 (44.4%) *FGFR3*^wt^/*TP53*^wt^, *n* = 4/18 (22.2%) *FGFR3*^mut^/*TP53*^wt^, *n* = 5/18 (27.8%) *FGFR3*^wt^/*TP53*^mut^ and *n* = 1/18 (5.6%) *FGFR3*^mut^/*TP53*^mut^.

**Figure 5:**
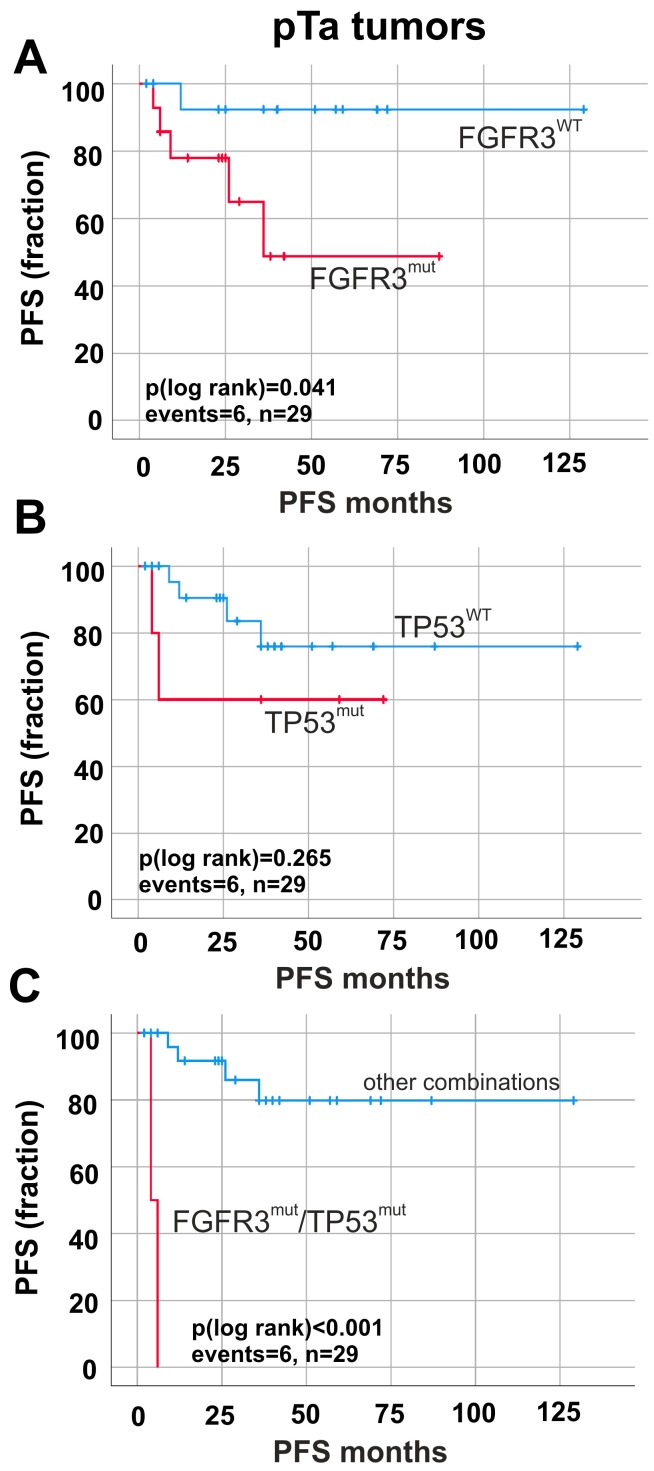
Prognostic impact of *FGFR3* and *TP53* mutations on tumor progression in papillary non-invasive (pTa) tumors. Progression-free survival (PFS) is shown. (**A**) Univariate survival analysis illustrates that detected *FGFR3* mutations (red curve, *n* = 14) predict shorter PFS compared to non-mutated *FGFR3* gene status (blue curve, *n* = 15). (**B**) Kaplan–Meier analysis of tumors with mutated *TP53* (red curve, *n* = 5) compared to wildtype *TP53* (blue curve, *n* = 24). (**C**) Impact of double mutations on risk stratification of tumor progression is shown. Survival analysis of double mutations (red curve, *n* = 2) compared to all other combinations of mutated and non-mutated *FGFR3* and *TP53* genes (blue curve, *n* = 27) in pTa tumors. *n*: overall number of cases; events: overall events of tumor progression.

Survival analyses revealed a correlation between *FGFR3* mutations and shorter PFS (*p* = 0.041) in pTa tumors (Figure 5A). *TP53* mutations did not show any effects (*p* > 0.05) on PFS (Figure 5B). Interestingly, tumors exhibiting double mutation status *FGFR3*^mut^/*TP53*^mut^ (*n* = 6) presented a dramatically reduced ∆mean PFS (*p* < 0.001) of 102.52 months (mean PFS: 5.00 months ± 1.00; 95% CI: 3.04 to 6.96) in pTa tumors (Figure 5C) compared with all other combinations (mean PFS: 107.52 months ± 9.72; 95% CI: 88.46 to 126.57).

Finally, we assessed the clinical impact of immunohistochemical and mutational status as a combined approach. Survival analysis displayed that *FGFR3*^mut^/*TP53*^mut^ double mutated tumors were significantly associated with worse patients’ outcome only in FGFR3 and Ki67 overexpressing tumors: reduced PFS in FGFR3^high^/Ki67^high^ double mutated tumors compared with all other combinations of molecular status of *TP53* and *FGFR3* (FGFR3^wt^/TP53^wt^ *p* = 0.001; FGFR3^mut^/TP53^wt^ *p* < 0.001; FGFR3^wt^/TP53^mut^ *p* = 0.116) (data not shown). However, it has to be noted that the number of double mutations is very low, and, hence, statistical validity should be enhanced in future studies.

### 2.5. FGFR3^high^/Ki67^high^ Tumors Define a Subset of pTa Tumors Including Lesions with Molecular FGFR3 Pathway Activation

Prognostic stratification of bladder cancer patients in routine histopathological diagnostics claims simple and cost-effective means, therefore, we evaluated the concordance of immunohistochemical staining results and molecular status.

The majority of FGFR3^high^/Ki67^high^ tumors was characterized by conjunct *FGFR3* mutations with a significant correlation only in papillary non-invasive tumors (pTa *n* = 19/27 (70.4%), *p* < 0.001) but not in pT1 and pT2–4 tumors (Table S5). There was no significant correlation between FGFR3^high^/Ki67^high^ tumors and *TP53* mutations independently of the given tumor stage (data not shown).

To evaluate the diagnostic potential of immunohistochemical markers covering the molecular FGFR3 pathway, we performed ROC (Receiver operating characteristics) curve statistics to calculate sensitivity and specificity. Accordingly, both immunohistochemical markers detect *FGFR3* mutations with 90.5% sensitivity and 61.9% specificity (area under curve (AUC): 0.776, *p* = 0.004, positive predictive value (PPV): 70.4%, negative predictive value (NPV): 85.7%). These data show that FGFR3^high^/Ki67^high^ tumors include papillary lesions with mutation-based altered FGFR3 signaling, but also tumors without molecular alterations (pTa *n* = 8/27 (29.6%)). Hence, our data give evidence that FGFR3^high^/Ki67^high^ tumors define a subset of pTa tumors associated with poor prognosis potentially decoupled from the described protective effect of FGFR3 activation [7].

## 3. Discussion

In our study, we systematically analyzed papillary non-invasive and invasive tumors for distinct prognostic immunohistochemical and molecular markers. We focused on a subgroup of immunohistochemically FGFR3^high^/Ki67^high^ tumors in order to reveal their prognostic impact on patient survival and re-evaluate their histological classification/grading.

Although, according to nuclear and architectural criteria, these papillary tumors appear to be orderly and more “nuclear low grade”, we found them associated with worse PFS compared with FGFR3^high^/Ki67^low^ tumors. This was especially evident in pTa tumors, where mean progression-free survival was reduced to 55 instead of 113 months. Therefore, we asked whether these tumors harbor a special molecular phenotype turning them into aggressive ones. In literature *FGFR3* and *TP53* mutations were initially thought to be mutually exclusive as *FGFR3* mutations were associated with pTa and LG tumors (“papillary pathway”), whereas the *TP53* mutations were often found in invasive and HG carcinomas (“CIS/invasive pathway”) [17,18]. Notwithstanding, Hernandez et al. reported *FGFR3* and *TP53* mutations to be independently distributed in a large series of pT1G3 tumors, that were consequently interpreted as a particular group of bladder tumors that could not be classified into either one pathway or the other [4]. In our study, we saw a similar trend with well-known inverse relationships between *FGFR3* and *TP53* mutations for both stage and grade, while mutations in *FGFR3* and *TP53* revealed an independent but not mutually exclusive assignment (six tumors with double mutations). Biologically activated FGFR3 signaling promotes cell proliferation and tumor growth, however interestingly, highest numbers of FGFR3-alterations are found in benign papillary or low grade papillary tumors with usually low proliferation (Ki67) index [19,20,21]. TP53 inactivation results in reduced cellular apoptosis and thus maintains tumor growth via reduced cell death [22,23,24,25]. We hypothesized that a FGFR3^high^/Ki67^high^ phenotype might be resulting from inactivated p53, however we found no sufficient molecular evidence for this theory in our cohort. Recent comprehensive sequencing data of papillary non-invasive bladder tumors revealed a genomic subtype 2, which is characterized by loss of 9q (including TSC1), increased Ki67 labeling index, upregulated mTORC1 signaling, glycolysis, features of the unfolded protein response, altered cholesterol homeostasis and DNA repair [15]. Therefore, high proliferation might be explained by mutations in DNA repair genes or the deletion/mutation of *TSC1*, which consequently leads towards an upregulation of mTORC1 and *PIK3CA* mutations. Further analyses to strengthen this theory have to be performed in the future.

Comprehensive molecular data of bladder cancer has been gained in the recent years [15,26,27], however, complex multigene analysis and RNA expression analysis are costly and laborious, and therefore cost-effective simple analyses for routine histological examination are needed. In our study, we analyzed whether fast and simple immunohistochemical analyses are suitable to detect a more aggressive molecular subtype. We found a highly significant correlation between strong FGFR3/Ki67 immunohistochemical staining and *FGFR3* mutation status, which indicates that FGFR3 protein expression is more frequent than mutational activation [16]. Moreover, our FGFR3^high^/Ki67^high^ subgroup also comprises those neoplasms without any molecular (*FGFR3* and/or *TP53*) alterations defining in this combination a subset of pTa tumors with poor prognosis, i.e., FGFR3 overexpression was associated with unfavorable outcome as previously shown, for instance, for invasive bladder tumors treated with adjuvant chemotherapy [28]. Thus, our data support the proposed clinical significance of these two immunohistochemical markers for diagnostic and prognostic stratification of more aggressive papillary non-invasive bladder tumors.

Taken together, we found immunohistochemically FGFR3^high^/Ki67^high^ pTa tumors associated with worse prognosis/survival, despite appearing histologically of “lower nuclear grade”/G2. Even if these tumors appear to be “low grade” (according to the 2004 WHO classification), we recommend classifying them as “high grade” pTa tumors. In light of our findings, we suggest immunohistochemical staining for FGFR3 and Ki67 in order to gain evidence for this more aggressive molecular subgroup with worse prognosis. These patients probably could profit from close endoscopic follow-up, as especially urine cytology might also be challenging/less sensitive due to their minimal nuclear changes.

## 4. Materials and Methods

### 4.1. Patient Samples, Tissue Microarrays and DNA

We retrospectively selected urothelial bladder cancer cases (mutational analysis: *n* = 42 pTa, *n* = 39 pT1, *n* = 18 pT2–4; immunohistochemical analysis: *n* = 82 pTa, *n* = 42 pT1, *n* = 18 pT2–4) from our pathology archive and from the archive of the Institute of Pathology in Erlangen. Formalin-fixed paraffin-embedded (FFPE) surgical specimens were used to construct tissue microarrays (all samples) and extract DNA (*n* = 99 samples) using Qiagen kits (Qiagen, Hilden, Germany) as previously described [29,30,31]. Patient information was obtained by the Department of Urology and the local ethics committee approved a retrospective, pseudonymized study of archival tissues (RWTH EK 009/12). Histological tumor grade and stage was classified according to WHO 2004 classification [8].

### 4.2. Immunohistochemistry

For immunohistochemical stainings, TMA sections were pretreated with DAKO PT-Link heat induced antigen retrieval with Low pH (pH 6) or High pH (pH 9) Target Retrieval Solution (DAKO, Hamburg, Germany) and incubated for 30 min at room temperature with respective antibodies in a DAKO Autostainer (DAKO). For stainings anti-FGFR3 (clone B9, PTlink pH6, dilution 1:25, Flex+M; Santa Cruz Biotechnology, Heidelberg, Germany), anti-Ki67 (clone MIB-1, PTlink pH 6, dilution 1:400, Flex+M; DAKO), anti-CK 20 (clone Ks20.8, PTlink pH 6, dilution 1:200, Flex+M; DAKO), and anti-CK5/6 (clone D5/16 B4, PTlink pH 9, dilution 1:100, Flex+M; DAKO) were used. Appropriate linker molecules EnVision^TM^FLEX+ (mouse/rabbit), EnVision FLEX/HRP detection system and counterstaining with EnVision FLEX Hematoxylin were applied. Stainings were evaluated by an experienced uropathologist (NTG) who was blinded for patient identity, diagnosis and clinical follow-up results. FGFR3 positivity was reported according to a semiquantitative scoring system developed by Tomlinson et al. [16]. All other stainings were evaluated for staining intensities (0 = no staining, 1 = weak staining, 2 = moderate staining, 3 = strong staining) and percentages of positive stained tumor cells. Results were judged as follows: Keratin 20 positive ≥10% stained cells [14,32], Keratin 5/6 positive ≥10% [32], Ki67 positive ≥15% [11,13] stained cells.

### 4.3. Fluorescence In Situ Hybridization

*ZytoLight* Dual Color Probe SPEC FGFR3/*CEN* 4 and *ZytoLight* Dual Color Break Apart Probe SPEC FGFR3 (Zytovision, Bremerhaven, Germany) were hybridized onto 3 µm TMA sections according to the manufacturer’s protocols. Slides were evaluated with a Zeiss Axiovert 135 fluorescence microscope (Carl Zeiss, Oberkochen, Germany), and Diskus Software (MIL 7.5, 4.80) (Büro Hilgers, Königswinter, Germany) using appropriate channels/filters (AHF ZyGreen F36-720, AHF ZyOrange F36-740, AHF DAPI, AHF F56-700). Signals of 60 nuclei of tumor cells were counted at high magnification (×1000) and judged as described previously [33].

### 4.4. Sanger Sequencing

PCR-amplification of exons 7, 10 and 15 of the *FGFR3* gene and exons 5, 6, 7, 8 and 9 of *TP53* were carried out using routine protocols. Primers and annealing temperatures are given in Table S6. PCR products were purified by either ExoSAP-IT (Affymetrix, Lahr/Schwarzwald, Germany) or a PCR purification kit (PerkinElmer Chemagen, Baesweiler, Germany) according to the manufacturer’s instructions. Sanger sequencing of both strands was run on an ABI PRISM 3500 Genetic Analyzer (Applied Biosystems, Weiterstadt, Germany) using the Big dye Terminator kit (Applied Biosystems), the same primer sets and the seq purification kit (PerkinElmer Chemagen).

### 4.5. Statistical Analysis

Statistical analysis was performed using SPSS (Statistical Package for the Social Sciences) software version 23.0 (SPSS Inc., Chicago, IL, USA). *p*-values < 0.05 were considered significant. Statistical associations between clinico-pathological and molecular factors were determined by Fisher’s exact test. Correlation analysis was performed by calculating a Spearman’s rank correlation coefficient. Survival (progression-free survival (PFS)) was calculated using the Kaplan–Meier method with log-rank statistics. Survival was measured from surgery until relapse, death or progression and was censored for patients alive without evidence of event at the last follow-up date. Multivariate Cox-regression analysis was performed to test for an independently prognostic value of FGFR3-Ki67 protein expression and *FGFR3-TP53* mutations. Receiver operating characteristics (ROC) curves were calculated to assess biomarker performance of immunohistochemical markers regarding molecular alterations.

## Figures and Tables

**Table 1:** Clinico-pathological parameters in correlation to FGFR3 protein expression.

	FGFR3 Expression ^a^				
	*n*	0–2	3	*p*-Value ^b^	Spearman ρ
Parameter:					
Age at diagnosis					
<70 years	67	20	47	0.946	0.006
≥70 years	75	22	53		
Gender					
female	31	10	21	0.372	0.031
male	111	32	79		
Histological tumor grade					
low grade	49	3	46	**<0.001**	−0.373
high grade	93	39	54		
Tumor stage					
pTa	82	14	68	**<0.001**	−0.320
pT1–pT4	60	28	32		

^a^ Tomlinson score according to [16]; ^b^ Fisher’s exact test; Significant *p*-values are marked in bold face.

**Table 2:** Clinico-pathological parameters in correlation to Ki67 protein expression.

	Ki67 Expression ^a^				
	*n*	<15%	≥15%	*p*-Value ^b^	Spearman ρ
Parameter:					
Age at diagnosis					
<70 years	67	31	36	0.083	0.146
≥70 years	75	24	51		
Gender					
female	31	14	17	0.408	0.070
male	111	41	70		
Histological tumor grade					
low grade	49	34	15	**<0.001**	0.457
high grade	93	21	72		
Tumor stage					
pTa	82	37	45	0.069	0.175
pT1–pT4	60	18	42		

^a^ According to [11]; ^b^ Fisher’s exact test; Significant *p*-values are marked in bold face.

**Table 3:** Multivariate Cox regression analysis of immunohistochemical markers including all factors potentially influencing PFS.

Variable	HR	*p*-Value	95%CI	
			Lower	Upper
FGFR3 ^high^/Ki67^high^	3.943	**0.019**	1.247	12.466
pT status	0.957	0.941	0.295	3.105
Tumor grade	0.846	0.823	0.196	3.653
Keratin 5/6	0.482	0.280	0.128	1.812
Keratin 20	0.424	0.115	0.146	1.232
Age	1.773	0.347	0.537	5.847

**Table 4:** Multivariate Cox regression analysis of molecular markers including all factors potentially influencing PFS.

Variable	HR	*p*-Value	95%CI	
			Lower	Upper
*FGFR3*^mut^/*TP53*^mut^	6.563	**0.006**	1.694	25.425
pT status	1.179	0.821	0.284	4.896
Tumor grade	0.241	0.138	0.037	1.580
Keratin 5/6	0.714	0.621	0.188	2.712
Keratin 20	0.872	0.814	0.279	2.730
Age	1.41	0.584	0.412	2.809

## Supplementary Materials

Supplementary materials can be found at http://www.mdpi.com/1422-0067/19/9/2548/s1.
