# Supplementary material for: Diagnostic and Prognostic Implications of FGFR3high/Ki67high Papillary Bladder Cancers
Source: Int J Mol Sci. 2018 Aug 28;19(9):2548. doi: 10.3390/ijms19092548 (PMC6163244; doi:10.3390/ijms19092548)
Supplement: Supplementary file 1 [file ijms-19-02548-s001.zip › Supplementary Figures S1.docx]

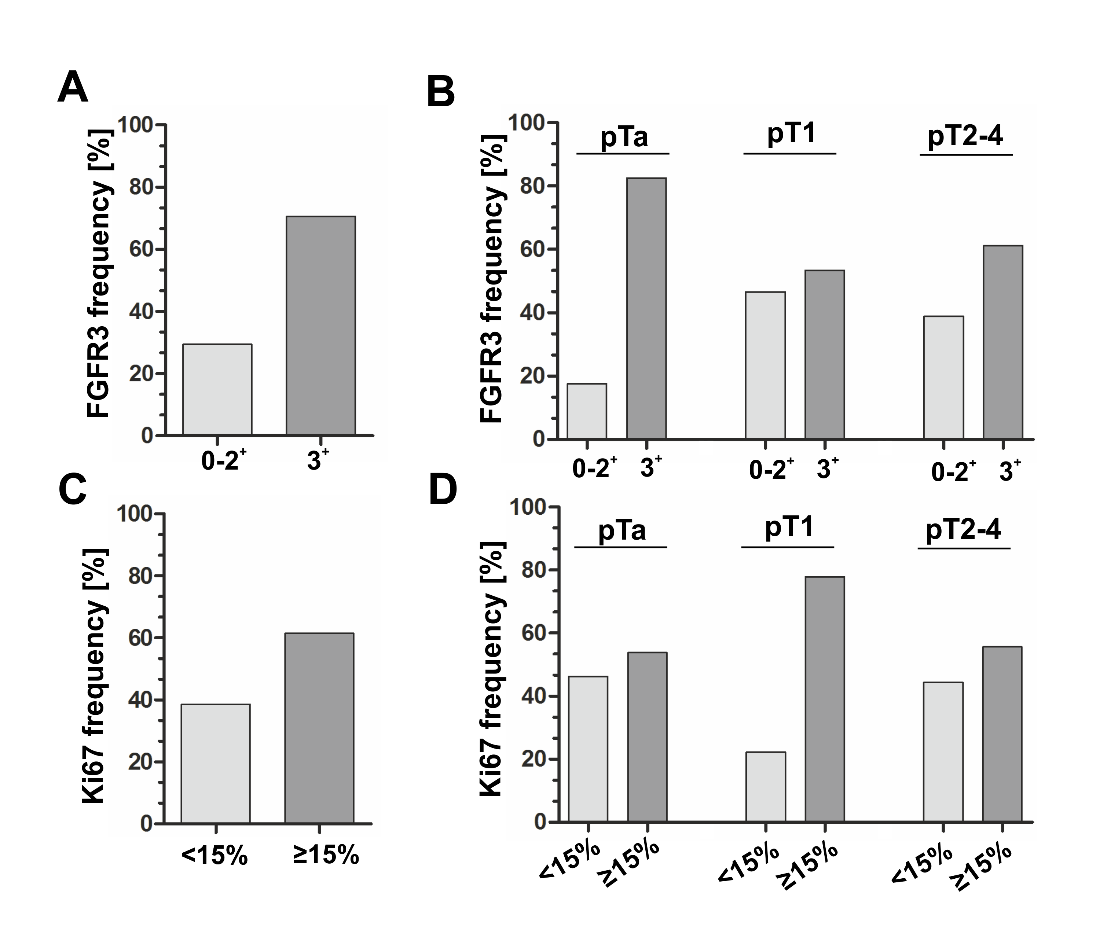


**Figure S1:** Frequency of FGFR3 and Ki67 protein expression in pTa, pT1 and pT2–4 bladder tumors**.** **(A)** Graph illustrating FGFR3 protein expression divided into two groups (low and moderate expression: Tomlinson Score 0–2; and overexpression: Tomlinson Score 3) in all tumors independently of a given subgroup. *Tomlinson Score **(B)** FGFR3 protein expression classified by pTa, pT1 and pT2–4 tumors. **(C)** Ki67 protein expression of nuclei dichotomized into two groups (low and moderate expression: <15%; and overexpression: ≥15%) in all tumors independently of a given subgroup. **(D)** Ki67 protein expression classified by pTa, pT1 and pT2–4 tumors.
