# Supplementary material for: Diagnostic and Prognostic Implications of FGFR3high/Ki67high Papillary Bladder Cancers
Source: Int J Mol Sci. 2018 Aug 28;19(9):2548. doi: 10.3390/ijms19092548 (PMC6163244; doi:10.3390/ijms19092548)
Supplement: Supplementary file 1 [file ijms-19-02548-s001.zip › Supplementary Figures S2.docx]

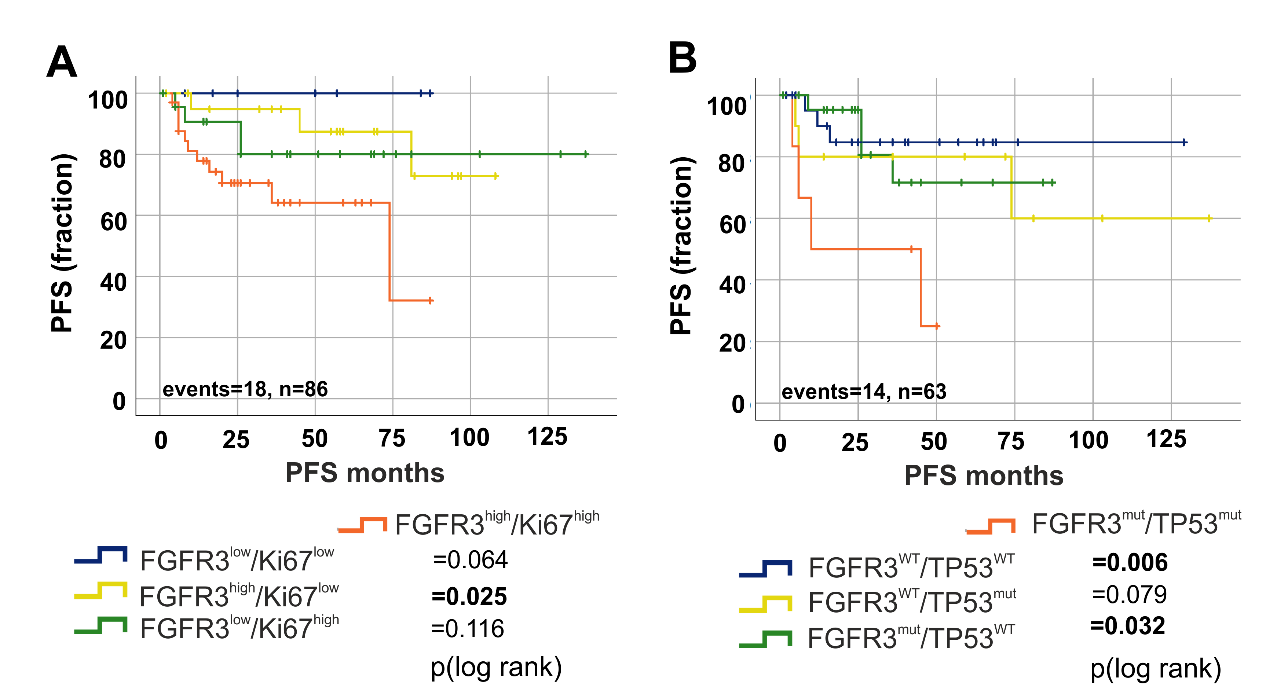


**Figure S2:** Prognostic impact of FGFR3 and Ki67 protein expression and FGFR3 and TP53 mutations stratified by groups in bladder cancer. Kaplan-Meier survival curves display progression-free survival (PFS). (A) Impact of combined markers (FGFR3 and Ki67) on risk stratification of tumor progression is shown. Survival curve analysis of FGFR3^high^/Ki67^high^ expression (orange curve) compared to all other combinations of FGFR3 and Ki67 expression. (B) Impact of combined molecular markers (*FGFR3* and *TP53*) on risk stratification of tumor progression is shown. Univariate analysis of double mutations (FGFR3^mut^/Ki67^mut^) compared to all other combinations of mutated and non-mutated *FGFR3* and *TP53* genes.
