# Supplementary material for: Diagnostic and Prognostic Implications of FGFR3high/Ki67high Papillary Bladder Cancers
Source: Int J Mol Sci. 2018 Aug 28;19(9):2548. doi: 10.3390/ijms19092548 (PMC6163244; doi:10.3390/ijms19092548)
Supplement: Supplementary file 1 [file ijms-19-02548-s001.zip › Supplementary Table 1.docx]

**Table S1.** Clinico-pathological parameters of 142 bladder cancer specimens analyzed in this study.

|  | **Categorization** | **n** | **% analyzable** |
| --- | --- | --- | --- |
|  |  |  |  |
| Parameter: |  |  |  |
| Age at diagnosis (median: 70) | <70 years | 67 | 47.2 |
|  | ≥ 70 years | 75 | 52.8 |
| Gender |  |  |  |
|  | female | 31 | 21.8 |
|  | male | 111 | 78.2 |
| Histological tumor grade |  |  |  |
|  | low grade | 49 | 34.5 |
|  | high grade | 93 | 65.5 |
| Tumor stage | |  |  |
|  | pTa | 82 | 57.7 |
|  | pT1 | 42 | 29.6 |
|  | pT2 | 10 | 7.0 |
|  | pT3 | 4 | 2.8 |
|  | pT4 | 4 | 2.8 |
