# Supplementary material for: Diagnostic and Prognostic Implications of FGFR3high/Ki67high Papillary Bladder Cancers
Source: Int J Mol Sci. 2018 Aug 28;19(9):2548. doi: 10.3390/ijms19092548 (PMC6163244; doi:10.3390/ijms19092548)
Supplement: Supplementary file 1 [file ijms-19-02548-s001.zip › Supplementary Table 2.docx]

**Table S2.** List of FGFR3 and TP53 mutations detected in this study.

|  |  | pTa | pT1 | pT2-4 |
| --- | --- | --- | --- | --- |
| FGFR3 | no. samples analyzed | 42 | 39 | 18 |
|  | samples with FGFR3 mutation | 21/42  (50%) | 22/39 (56.4%) | 5/18 (27.8%) |
|  | single mutations | 20/21 (95.2%) | 20/22 (90.9%) | 5/5  (100%) |
|  | multiple alterations | 1/21  (4.8%) | 2/22  (9.1%) | / |
|  | p.S249C | 13/21 (61.9%) | 9/22  (40.9%) | 2/5  (40.0%) |
|  | p.Y373C | 2/21  (9.5%) | 2/22  (9.1%) | 1/5  (20%) |
|  | p.K650E | 2/21  (9.5%) | 2/22  (9.1%) | / |
|  | p.R248C | 3/21  (14.3%) | 7/22  (31.8%) | 2/5  (40%) |
|  | p.G380R  +  p.K650N | 1/21  (4.8%) | / | / |
|  | p.R248C  +  p.S249C | / | 1/22  (4.5%) | / |
|  | p.R248C  +  p.G380R | / | 1/22  (4.5%) | / |
| TP53 | no. samples analyzed | 42 | 38 | 18 |
|  | samples with TP53 mutation | 6/42 (14.3%) | 11/38 (28.9%) | 6/18 (33.3%) |
|  | single mutations | 5/6  (83.3%) | 7/11  (63.6%) | 6/6  (100%) |
|  | missense | 5/6  (83.3%) | 6/11 (54.5%) | 5/6  (83.3%) |
|  | nonsense | / | 1/11  (9.1%) | / |
|  | frameshift | / | / | 1/6  (16.7%) |
|  | multiple alterations | 1/6  (16.7 %) | 4/11  (36.4%) | / |
|  | multiple missense | 1/6  (16.7%) | 1/11  (9.1%) | / |
|  | missense  +  nonsense | / | 2/11  (18.2%) | / |
|  | missense  +  in-frame deletion | / | 1/11  (9.1%) | / |
