# Supplementary material for: Diagnostic and Prognostic Implications of FGFR3high/Ki67high Papillary Bladder Cancers
Source: Int J Mol Sci. 2018 Aug 28;19(9):2548. doi: 10.3390/ijms19092548 (PMC6163244; doi:10.3390/ijms19092548)
Supplement: Supplementary file 1 [file ijms-19-02548-s001.zip › Supplementary Table 3.docx]

**Table S3:** Clinico-pathological parameters in relation to FGFR3 mutations.

|  | **FGFR3 mutations** | | | |  |
| --- | --- | --- | --- | --- | --- |
|  | ***n*** | **WT** | **Mut** | **P-value**^a^ | **Spearman ρ** |
| Parameter | | | |  |  |
| Age at diagnosis |  |  |  |  |  |
| <70 years | 42 | 26 | 16 | 0.077 | 0.178 |
| ≥70 years | 57 | 25 | 32 |  |  |
| Gender | | | |  |  |
| female | 18 | 13 | 5 | 0.053 | 0.195 |
| male | 81 | 38 | 43 |  |  |
| Histological tumor grade |  |  |  |  |  |
| low grade | 12 | 4 | 8 | 0.181 | -0.135 |
| high grade | 87 | 47 | 40 |  |  |
| Tumor stage |  |  |  |  |  |
| pTa | 42 | 21 | 21 | 0.797 | -0.026 |
| pT1-pT4 | 57 | 30 | 27 |  |  |

**^a^**Fisher’s exact test; Significant P-values are marked in bold face.
