# Supplementary material for: Diagnostic and Prognostic Implications of FGFR3high/Ki67high Papillary Bladder Cancers
Source: Int J Mol Sci. 2018 Aug 28;19(9):2548. doi: 10.3390/ijms19092548 (PMC6163244; doi:10.3390/ijms19092548)
Supplement: Supplementary file 1 [file ijms-19-02548-s001.zip › Supplementary Table 4.docx]

**Table S4..C**linico-pathological parameters in relation to TP53 mutations.

|  | **TP53 mutations** | | | |  |
| --- | --- | --- | --- | --- | --- |
|  | ***n*** | **WT** | **Mut** | **P-value**^a^ | **Spearman ρ** |
| Parameter | | | |  |  |
| Age at diagnosis |  |  |  |  |  |
| <70 years | 42 | 33 | 9 | 0.681 | 0.042 |
| ≥70 years | 56 | 42 | 14 |  |  |
| Gender | | | |  |  |
| female | 18 | 12 | 6 | 0.277 | -0.110 |
| male | 80 | 63 | 17 |  |  |
| Histological tumor grade^c^ |  |  |  |  |  |
| low grade | 12 | 12 | 0 | **0.042** | 0.207 |
| high grade | 86 | 63 | 23 |  |  |
| Tumor stage^d^ |  |  |  |  |  |
| pTa | 42 | 36 | 6 | 0.065 | 0.188 |
| pT1-pT4 | 56 | 39 | 17 |  |  |

**^a^**Fisher’s exact test; Significant P-values are marked in bold face.
