# Supplementary material for: Diagnostic and Prognostic Implications of FGFR3high/Ki67high Papillary Bladder Cancers
Source: Int J Mol Sci. 2018 Aug 28;19(9):2548. doi: 10.3390/ijms19092548 (PMC6163244; doi:10.3390/ijms19092548)
Supplement: Supplementary file 1 [file ijms-19-02548-s001.zip › Supplementary Table 5.docx]

|  | | **FGFR3 mutation** | | | |  |
| --- | --- | --- | --- | --- | --- | --- |
|  | | ***n*** | **WT** | **Mut** | **P-value**^a^ | **Spearman ρ** |
|  | | | | |  |  |
| pTa tumors | |  |  |  |  |  |
|  | FGFR3 ^other^ Ki67 ^other^ | 15 | 13 | 2 | **<0.001** | 0.547 |
|  | FGFR3 ^high^ Ki67 ^high^ | 27 | 8 | 19 |  |  |
| pT1 tumors | |  |  |  |  |  |
|  | FGFR3 ^other^ Ki67 ^other^ | 24 | 12 | 12 | 0.233 | 0.164 |
|  | FGFR3 ^high^ Ki67 ^high^ | 15 | 5 | 10 |  |  |
| pT2-4 tumors | |  |  |  |  |  |
|  | FGFR3 ^other^ Ki67 ^other^ | 11 | 7 | 4 | 0.322 | -0.240 |
|  | FGFR3 ^high^ Ki67 ^high^ | 7 | 6 | 1 |  |  |

**Table S5:** Correlation of FGFR3 / Ki67 protein expression with FGFR3 mutations.

^a^Fisher’s exact test; Significant P-values are marked in bold face.
