# Supplementary material for: Diagnostic and Prognostic Implications of FGFR3high/Ki67high Papillary Bladder Cancers
Source: Int J Mol Sci. 2018 Aug 28;19(9):2548. doi: 10.3390/ijms19092548 (PMC6163244; doi:10.3390/ijms19092548)
Supplement: Supplementary file 1 [file ijms-19-02548-s001.zip › Supplementary Table 6.docx]

| **primer** | **sequence 5’ – 3’** | **annealing temperature**  **°C** |
| --- | --- | --- |
| FGFR3 exon 7F | 5’ AGT GGC GGT GGT GGT GAG GGA G 3’ | 63 |
| FGFR3 exon 7R | 5’ TGT GCG TCA CTG TAC ACC TT 3’ |  |
| FGFR3 exon 10F | 5’ GCC AGG CCA GGC CTC AAC 3’ | 65 |
| FGFR3 exon 10R | 5’ CTT GAG CGG GAA GCG GGA GAT CTT G 3’ |  |
| FGFR3 exon 15F | 5’ TGG TGA CCG AGG ACA ACG TGA TG 3’ | 63 |
| FGFR3 exon 15R | 5’ CTC TGG TGA GTG TAG ACT CG 3’ |  |
| TP53 exon 5F | 5’ TGC CGT CTT CCA GTT GCT TTA TC 3’ | 60 |
| TP53 exon 5R | 5’ GCA ATC AGT GAG GAA TCA GAG GC 3‘ |  |
| TP53 exon 6F | 5’ AGC AGC TGG GGC TGG AGA G 3’ | 63 |
| TP53 exon 6R | 5’ CTG GAG GCC CAC TGA CAA C 3’ |  |
| TP53 exon 7F | 5’ CCA AGG CGC ACT GGC CTC A 3’ | 63 |
| TP53 exon 7R | 5’ AGA GGC AAG CAG AGG CTG G 3’ |  |
| TP53 exon 8F | 5’ CTG ATT TCC TTA CTG CCT C 3’ | 60 |
| TP53 exon 8R | 5’ CTG CAC CCT TGG TCT CCT C 3’ |  |
| TP53 exon 9F | 5’ GTT ATG CCT CAG ATT CAC TT 3’ | 55 |
| TP53 exon 9R | 5’ CGG CAT TTT GAG TGT TAG AC 3’ |  |

**Table S6.** Sequences of all primers used in this study for Sanger sequencing.
